# Antidepressant Effect of Ketamine on Inflammation-Mediated Cytokine Dysregulation in Adults with Treatment-Resistant Depression: Rapid Systematic Review

**DOI:** 10.1155/2022/1061274

**Published:** 2022-09-16

**Authors:** Shiryn D. Sukhram, Grozdena Yilmaz, Jianying Gu

**Affiliations:** Department of Biology, City University of New York-College of Staten Island, Staten Island, NY 10314, USA

## Abstract

**Background:**

Major depressive disorder (MDD) and treatment-resistant depression (TRD) represent a global source of societal and health burden. To advise proper management of inflammation-related depression among TRD patients, it is important to identify therapeutic clinical treatments. A key factor is related to proinflammatory cytokines such as interleukin- (IL-) 1*β*, IL-6, and tumor necrosis factor- (TNF-) *α* which have been implicated in the pathogenesis of depressive symptoms in MDD patients. Ketamine may provide an anti-inflammatory therapeutic strategy by targeting proinflammatory pathways associated with depressive disorders, which may be exacerbated in the ageing population with TRD.

**Objective:**

Despite a burgeoning body of literature demonstrating that inflammation is linked to TRD, there is still a lack of comprehensive research on the relationship between proinflammatory biomarkers and ketamine's antidepressant effect on TRD patients.

**Method:**

The Cochrane Library and PubMed/MEDLINE databases were systematically searched from inception up to February 1, 2022, adopting broad inclusion criteria to assess clinical topics related to the impact of ketamine on inflammatory cytokines in TRD patients. The present work is in compliance with the World Health Organization Rapid Review Guide.

**Results:**

Five out of the seven studies examined in this review show that ketamine infusion may reduce depressive symptoms with a quick start of effect on TRD patients. Based on the Montgomery-Åsberg Depression Rating Scale (MADRS) and Hamilton Depression Rating Scale (HAM-D) scores, the overall response rate for ketamine was 56%; that is, 56% of those treated with ketamine had MADRS/HAM-D scores decreased by at least 50%.

**Conclusions:**

While the anti-inflammatory effects of ketamine modulate specific proinflammatory cytokines, its rapid antidepressant effect on TRD patients remains inconsistent. However, our study findings can provide a reliable basis for future research on how to improve systemic inflammatory immune disorders and mental health. We suggest that ketamine infusion may be part of a comprehensive treatment approach in TRD patients with elevated levels of depression-specific inflammatory biomarkers.

## 1. Introduction

Depression has been recognized as a pandemic [1], and its etiology remains largely unknown. Major depressive disorder (MDD) is a mental health disorder that clinically presents itself through changes in mood and perception as well as a loss of pleasure lasting at least two weeks [2]. Moreover, MDD is set to become the leading cause of disability worldwide by the year 2030 [3], and approximately one-third of patients with MDD have treatment-resistant depression (TRD) [4]. Patients with TRD are typically identified as a failure to achieve remission after two or more trials of appropriate pharmaceutical treatment for MDD of a suitable dose and duration. However, a universally recognized classification of TRD is lacking in the scientific literature. In a recent publication, Zhdanava et al. [5] evaluated the prevalence and national burden of TRD and MDD in the United States. The authors reported that the estimated 12-month prevalence of medication-treated MDD in the U.S. was 8.9 million adults, and 2.8 million (30.9%) had TRD. There is a significant prevalence of TRD patients with elevated inflammatory markers, and this could be helpful in intervention strategies that can decrease their effects. The COVID-19 Mental Disorders Collaborators [6] recently published a systematic review on the mental health effects of the COVID-19 pandemic on the prevalence of depressive and anxiety disorders. The authors reported that the COVID-19 pandemic led to an additional 10.7 million (7.21–14.9) disability-adjusted life years (DALYs) for MDD globally, of which 7.07 million (4.80–9.80) were among women and 3.62 million (2.40–5.09) were among men. Furthermore, a recent World Health Organization (WHO) [7] brief indicated that the pandemic particularly affected the mental health of young people, consequently putting them at a disproportionate risk of suicidal and self-harming actions. The WHO brief further states that individuals with preexisting physical health conditions were more likely to develop symptoms of mental disorders.

**Figure 1:**
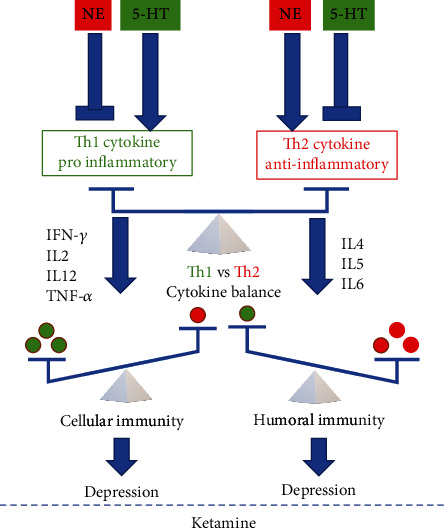
Schematic figure illustrating the putative targets of ketamine and its antidepressant effect on systemic regulators of the adaptive immune system. Adapted from [10]. Copyright by the Multidisciplinary Digital Publishing Institute.

The role of proinflammatory cytokines in depression has been investigated over the past decades [8], and recently, ketamine has been identified for therapeutic interventions. Previous animal studies have indicated ketamine's strong anti-inflammatory effect and propose cytokines as effective molecular biomarkers to examine ketamine's antidepressant effect [9]. According to clinical studies, ketamine's antidepressant effect may play a role in peripheral and central immune regulations [10, 11]. Ketamine may potentially lower cytokine levels associated with inflammatory processes, related to mood problems, or improve communication within certain brain regions [12]. It is hypothesized that the antidepressant treatment effect of ketamine may be associated with the ability of anti-inflammatory mediators to suppress proinflammatory mediators, such as IL-1*β*, IL-6, and TNF-*α* [13].  Figure 1 illustrates the putative targets of ketamine and its antidepressant effect on systemic regulators of the adaptive immune system. Disruptions of the Th1/Th2 immune ratio are associated with the pathophysiology of MDD [10]. Norepinephrine (NE) and 5-hydroxytryptamine (5-HT) present differential and distinct effects on inflammation and facilitate a Th1 shift and a Th2 shift, respectively, resulting in depressive symptoms. Hashimoto [10] indicates that NE inhibits the production of Th1 proinflammatory cytokines, such as TNF-*α*, whereas 5-HT inhibits the production of Th2 cytokines, such as IL-6. Also, a Th2 shift causes inflammation and an increase in proinflammatory cytokines consequently triggering depression. Ketamine may induce a Th1 and Th2 shift, respectively. A systemic protective anti-inflammatory effect is typically initiated when an individual faces a localized inflammatory reaction, specifically a predominance of the Th2 (anti-inflammatory) upon the Th1 (proinflammatory) pathway [14]. De Kock et al. [14] indicate that ketamine performs as a distinctive homeostatic regulator of the acute inflammatory response and associated stress-induced conditions. The administration of a single IV dose of ketamine (0.5 mg/kg) significantly interferes with this immune ratio and increases the Th1/Th2 ratio which may be advantageous to an individual's global immune function and overall health [14, 15]. Therefore, the regulation of the “immune balance” between Th1-mediated cellular immunity and Th2-mediated humoral immunity is essential for therapy and management of TRD.

Ketamine can suppress proinflammatory cytokine levels, and decreases in serum levels strongly correspond with the relief of depressive symptoms [13]. Emerging data suggest that ketamine delivers its positive antidepressant effect in a dose-dependent way through IL-6 blockage [16]. However, the use of various proinflammatory cytokines in ketamine intervention strategies warrants further investigation. The field of psychoneuroimmunology has established a significant relationship between immunological processes and mental disorders [17]. Increasing evidence proposes a reciprocal relationship between low-grade systemic inflammation and MDD, suggesting that depression is an inflammatory disease. Upregulation of inflammatory markers has been linked to MDD involving induction of a proinflammatory state. As described by Dinarello [18], proinflammatory cytokines act to make the disease worse while anti-inflammatory cytokines work to reduce inflammation and stimulate healing. A compelling body of evidence has emerged in recent years suggesting the role of inflammatory cytokines in the pathophysiology of MDD [19]. A distinct pattern of inflammatory cytokine concentrations in patients with MDD might explain their manifestation or prognosis mediated by IL-1 or IL-6 [20]. Additionally, several studies have shown that proinflammatory cytokine levels are increased in patients with inflammatory- and age-related processes. A significant cytokine associated with depression in the elderly is related to IL-1*β*. For instance, IL-1*β* levels were elevated in depressed elderly subjects older than 60 years, directly relative to the severity of disease [21]. A thorough understanding regarding the impact of ketamine treatment on IL-1*β* may lead to novel treatment strategies for the ageing population [22].

Early and appropriate clinical treatment directed at proinflammatory cytokines may reduce the occurrence of depression-like symptoms commonly observed in TRD patients. Recent research findings indicate that intravenous (IV) ketamine infusion may potentially lower serum inflammatory cytokine levels associated with TRD. However, the limited studies available are inconsistent when reporting the antidepressant effect of ketamine on proinflammatory cytokines. The goal of the present report is to explore the available evidence and the existing gaps concerning the implications of ketamine treatment among dysregulated cytokine mediators in TRD.

While clinical researchers have yet to agree on a coherent explanation, individuals with dysregulated immune responses should be cautious of the prospective influence their condition could have on their mental health. According to the WHO [7], the COVID-19 pandemic has had negative effects on the mental health of the global population, including higher rates of suicidal ideation and suicide attempts. Inflammation induced by COVID-19 may contribute to the increased risk for depression. Furthermore, as the population ages, it is important to identify therapeutic strategies for depressive symptoms associated with inflammatory processes. Late-life depression may present high pathogenic complexity caused by chronic illness and psychosocial/physiological problems compared to depression at a younger age [1, 22]. Statistical data from the United Nations [23] indicate that by 2050, one in six people in the world will be older than 65 (16%).

As the world's ageing population is expected to increase, there is a need to investigate whether anti-inflammatory therapeutic strategies targeting proinflammatory pathways could reduce mental morbidity in the future. A population, intervention, comparison, and outcome- (PICO-) related question was used to guide the systematic review: “What are the effects of ketamine infusion of 0.5mg/kg (I) on adult patients diagnosed with treatment-resistant depression (P) on depression scale scores and serum proinflammatory cytokine levels (O) compared to healthy controls, or placebo (C)?” The aim of this rapid systematic review is to evaluate (a) the relationship between the antidepressant effect of ketamine and proinflammatory IL-1*β*, IL-6, and TNF-*α* cytokine response, (b) the impact of ketamine treatment in the management of inflammation-related TRD, and (c) knowledge gaps in these contexts. This manuscript is not intended to be a systematic review of the existing literature regarding the effectivity and/or management strategies of ketamine and instead is intended to provide relevant guidance to public health and policy-makers on strategies and interventions to improve TRD among individuals with inflammation-related depressive conditions. Systematic reviews providing a comprehensive overview of (1) the efficacy/tolerability/safety profile of ketamine as an equally effective treatment alternative to electroconvulsive therapy in TRD patients and (2) the effect of ketamine on the relationship between inflammation and glutamate signaling in depressive disorders are available elsewhere [11, 24–27]. However, there is still a lack of comprehensive research on the relationship between proinflammatory biomarkers and ketamine's antidepressant effect. This minisystematic review is aimed at presenting ketamine-induced effects on proinflammatory IL-1*β*, IL-6, and TNF-*α* cytokines. The guidance provided herein is based on clinical studies relevant to the antidepressant effect of ketamine on inflammation-mediated cytokines in adult TRD patients.

## 2. Methods

**Figure 2:**
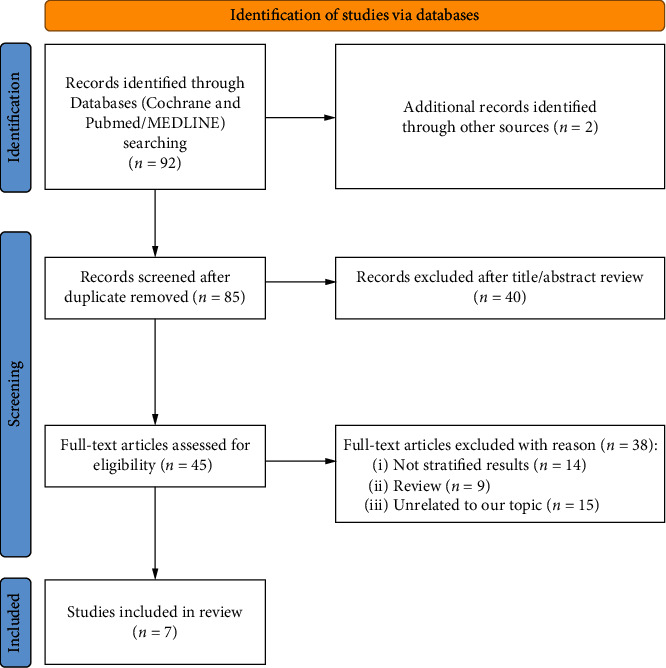
Study flow based on the 2020 flow diagram for new systematic review which included search of databases and registers only. Adapted from [29]. Copyright by the British Medical Association.

This rapid review was completed in accordance with the World Health Organization Rapid Review Guide [28]. Compliance with the WHO guidelines indicates methodological reliability and application of research findings across rapid reviews. Comprehensive systematic searches were conducted in the Cochrane Library and PubMed/MEDLINE databases from inception up to February 1st, 2022. The query was conducted with the following terms (in all fields): (“ketamine” AND “depression” AND “cytokine”). We also scanned the reference lists of all included articles for studies to be included in our work. The Preferred Reporting Items for Systematic Reviews and Meta-Analyses (PRISMA) 2020 flow diagram was used to illustrate the extraction of studies through the different phases of this review [29]. Please refer to Figure 2 for details about the adopted search strategy.

Two investigators (SS and GY) screened the retrieved references for eligibility both at the title/abstract and full-text levels. Disagreements were resolved by discussion or by the judgement of the third reviewer (JG). During the review process, studies were only included in the next phase of analysis upon the agreement of at least two reviewers. One reviewer obtained the data, and the findings of these extractions were thoroughly reviewed by the coauthors.

Key elements extracted from the literature included (1) first author, (2) year of publication, (3) country of origin, (4) study population and sample size, (5) methodology, (6) gender ratio, (7) average age, (8) intervention type and comparator (if applicable), (9) duration of the intervention (if applicable), (10) diagnostic test or criteria, and (11) key findings that relate to the review question.

We excluded non-English articles, unavailable full-text articles, animal studies, case reports, case studies, opinions, editorials, commentaries, letters, conference abstracts, preclinical studies, and reviews. Criteria for depression had to be established by a coded diagnosis according to the Diagnostic and Statistical Manual of Mental Disorders (DSM) criteria. After exclusion of duplicates, we screened the title/abstract or full-text reports and decided whether these met the inclusion criteria.

We included experimental studies (randomized—individually or cluster—and nonrandomized controlled trials) and observational studies with an internal comparison group (cohort—prospective and retrospective—and case-control) studies. Articles were included based on the following criteria: (a) written in English; (b) included any measure assessing ketamine, depression, and cytokine levels; and (c) qualitatively examined and presented results of the relationship between ketamine and cytokine-related outcomes (e.g., correlations). Notably, to be included in this systematic approach, it was not required for studies to test the association between TRD and proinflammatory cytokine levels; for instance, if a serum cytokine level and mental-related outcome variables were both included in a correlation matrix, the study was included.

## 3. Results

### 3.1. General Characteristics

**Figure 3:**
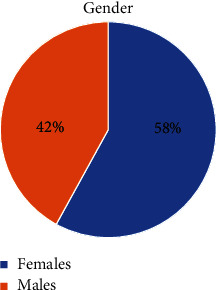
Gender distribution for articles reporting on the antidepressant effect of ketamine on inflammatory cytokines in adult patients with treatment-resistant depression.

Seven studies which reported on the association of the antidepressant effect of ketamine infusion on serum inflammatory cytokine levels in TRD patients were included in this systematic review. The overall age distributions for the six studies which included demographic data [30–35] were calculated as combined means and standard deviation of age to get a weighted mean and pooled standard deviation, which results in age distribution of 41.298 ± 11.74 (95%Confidence Interval (CI) = [18.29279, 64.30407]); thus, approximately 50% of the subjects are more than likely >40 years. The overall gender distribution was calculated by adding mean percentages which resulted in 58% female (Figure 3). Yang et al. [36] lack any demographic data; therefore, we only included studies that reported age and gender frequencies to calculate the overall age and gender distribution for this review. These relative estimates along with the key findings of their associated studies are summarized in Table 1.

According to six out of seven studies, this review examined 200 females and 159 males with TRD. Kruse et al.'s study [32] indicate that inflammatory profiles, depressive symptoms, and depression treatment response differ between females and males. Statistical analyses stratified by gender revealed that increasing IL-8 was associated with a decreasing Hamilton Depression Rating Scale (HAM-D) score in females (*p* = 0.08), while the inverse was found in males (*p* = 0.02). Gender-specific processes trigger the neural and/or behavioral effects of IL-8; however, further statistical analysis stratified by gender did not illustrate a differential change between ketamine treatment outcome and CRP, IL-6, IL-10, and TNF-*α* levels [32]. Our findings indicate no statistically significant relationship between gender and ketamine response rates or mean age and ketamine response rate, i.e., no associations were found between the number of females and how many responded positively.

### 3.2. Treatment-Resistant Depression (Population)

In each of the studies reviewed, recurrent MDD was diagnosed according to the DSM criteria. The DSM criteria published by the American Psychiatric Association have been widely used by clinicians to diagnose mental health disorders. However, various versions of the DSM were utilized in the studies. The DSM has been revised seven times since it was first published in 1952 [37]. Kruse et al. [32] was the only study that did not report which DSM version was used for MDD diagnosis. Kiraly et al. [31] and Yang et al. [36] utilized the DSM-IV criteria. The DSM-IV-TR was published in 2000 and used in the studies done by Chen et al. [30] and Park et al. [33]. The revised DSM-IV-TR version classified disorders using a multiaxial or multidimensional approach to ensure that biological, environmental, and psychological factors were considered when making a comprehensive mental health diagnosis. The DSM-V [38], published in 2013, was used in the studies examined by Zhan et al. [34] and Zhou et al. [35].

Reliability and consistency of clinical assessment for MDD based on the DSM criteria should remain consistent when diagnosing recurring MDD and accordingly TRD. There is no universally recognized classification of TRD, and consequently, it has been treated as a homogenous entity [39, 40]. In each of the studies reviewed, TRD was defined as MDD patients who had failed response to at least two different adequate antidepressant treatment trials. Additionally, treatment resistance occurs generally in approximately 30% of treated MDD patients [41]; therefore, improved TRD management is warranted that includes the effect of specific targeted treatment strategies encompassing psychological therapies. Kiraly et al. [31] and Park et al. [33] determined TRD by using the Antidepressant Treatment History Form (ATHF). The ATHF has been the most widely used instrument to systematically evaluate antidepressant treatment trials and identify treatment resistance [42].

### 3.3. Ketamine Infusion (Intervention)

Ketamine hydrochloride (PubChem CID: 15851) is typically administered via IV infusion therapy. Each of the studies [30–36] reviewed utilized a constant IV ketamine dose of 0.5 mg/kg. There was variability in the frequency of IV ketamine infusions. Overall, five studies reported a single-dose ketamine infusion of 40 minutes, and all five publications reported elevations in at least cytokine inflammatory markers which were concomitant with depressive symptoms [30–33, 36]. Chen et al. [30] is the only study that compared different dosages of ketamine infusions. The patients were assigned randomly in a 3 : 1 ratio and received either a ketamine infusion of 0.5 mg/kg or a ketamine infusion of 0.2 mg/kg or a saline placebo infusion. Patients with higher baseline IL-6 (OR: 8.93, 95% CI: 1.16–68.86) levels were significantly associated with treatment response in the 0.5 mg/kg ketamine group. These findings suggest that a higher dose (0.5 mg/kg) of ketamine exerts an anti-inflammatory effect, and a lower dose (0.2 mg/kg) of ketamine may be associated with causal factors other than inflammation.

Zhan et al. [34] and Zhou et al. [35] were the only studies that examined longer ketamine treatment duration. Zhan et al. [34] administered six IV ketamine infusions in a 12-day period (days 1, 3, 5, 8, 10, and 12) and examined cytokine levels at baseline and 24 hours and 14 days after the sixth infusion (days 0, 13, and 26). Approximately 58.3% patients met the treatment response criteria after six ketamine infusions (day 13), and 25 (41.7%) attained remission. Zhou et al. [35] presented a post hoc analysis of the antidepressant effect of six adjunctive ketamine infusions on TRD patients with and without the presence of painful symptoms. TRD patients received ketamine infusions three times weekly for 2 weeks, and inflammatory cytokine levels were examined at baseline, day 13, and day 26. In both the pain and nonpain groups, significant reductions in Montgomery-Åsberg Depression Rating Scale (MADRS) scores were found at 24 hours postinfusion compared to baseline scores, and these reductions were maintained over the subsequent infusion period as well as on day 26 (*p* < 0.05 for both groups).

### 3.4. Healthy Controls and/or Placebo (Comparison)

Kiraly et al. [31], Yang et al. [36], and Zhou et al. [35] were the only studies to include control subjects. It is normally assumed that a “healthy” control in a clinical study is defined as an individual who does not have the condition being investigated. However, a “healthy” control may have other underlying disorders that are not examined in the particular area of research. This leads to the question: how healthy are “healthy volunteers”? [43]. Twenty-six healthy volunteers in the study done by Kiraly et al. [31] were free of lifetime psychiatric conditions or major medical illnesses including active or systemic infections. Twenty-four matched healthy volunteers with no present or past history of any DSM-IV Axis I or Axis II diagnosis were examined by Yang et al. [36]. Inflammation plays a vital role in multiple disorders including depression, inflammaging, and pain, and the inclusion of reliable “healthy” controls with stricter criteria should be considered to prevent the risk of overestimating differences and possibly affecting the outcome of clinical studies. Zhou et al. [35] examined sixty healthy controls matched by age and gender.

Volunteer participation in clinical trials usually does not provide direct health benefits; however, participants are typically compensated with compensation dependent upon the duration of the study and clinical measures performed. Therefore, as discussed by Marchesini et al. [43], the terms “volunteer” and “normal range” also present difficulties in evidence-based medicine. Nevertheless, the inclusion of healthy controls is significant to ketamine research, as it provides comparison data when investigating the effects of treatments on individuals with specific disorders, especially related to inflammatory conditions.

### 3.5. Depression Scale Scores and/or Proinflammatory Cytokine Levels (Outcome)

After a basic statistical analysis of the studies included in the review, six papers reported response rates, defined as a ≥50% decrease in MADRS and HAM-D scores. The MADRS and HAM-D scales are commonly used in clinical studies to measure indicators of depression. Leucht et al. [44] compared the HAM-D and MADRS scores in a large sample of patients with MDD and proposed that the linking of the absolute change in HAM-D total scores with the MADRS total scores could be used in metastudies.

In this review, the findings of the HAM-D scores in Kruse et al.'s study [32] were pooled with the MADRS scores of other studies [31, 33–36]. The overall response rate for ketamine was 56% ± 8.41% (95%CI = [39.48635%, 72.45394%]); that is, 56% of those treated with ketamine had MADRS/HAM-D scores decrease by at least 50%. The response rates were reported 24 hours after treatment, except for Zhan et al. [34], which was only reported at 13 days after treatment. Note that for this analysis, patients who were healthy controls or placebos were excluded where relevant, i.e., did not receive ketamine. Response rates were available in Kiraly et al. [31], Kruse et al. [32], Zhan et al. [34], Zhou et al. [35], and Yang [36].

“Proinflammatory cytokines” was used as a comprehensive term that included at least one of the following proinflammatory mediators in the studies reviewed: IL-1*β*, IL-6, and TNF-*α*. It is noteworthy that IL-6 was the most characterized type of proinflammatory cytokine associated with TRD and consequent ketamine treatment efficacy. Regardless of increasing evidence in the literature, and as illustrated in this review (Table 1), the targeted regulation of cytokine IL-1*β* and TNF-*α* response for ketamine's antidepressant effect requires further investigation, especially with gender and characteristic age distribution.

The randomized controlled trial by Chen et al. [30] demonstrated that changes in TNF-*α* between 40 minutes and baseline, but not at 240 minutes, were positively correlated with changes in the MADRS across time; however, the significant relationship between TNF-*α* change and changes in MADRS scores was only noted for day 4 and day 5 (*p* < 0.05) but not for other days (*p* > 0.05). Furthermore, the findings indicated no significant changes in CRP levels between baseline and postinfusion (*p* = 0.472). The clinical trial by Kiraly et al. [31] reported that IL-6 and IL-1*α* levels, 4 hour postinfusion with ketamine, showed modest but statistically significant decreases from baseline (*p* < 0.05, *t* = 2.369 and *p* < 0.05, *t* = 2.149, respectively); however, the levels of all inflammatory cytokines investigated IL-6, IL-1*α*, IL-1*β*, and TNF-*α* were no longer significantly different from baseline to 24 hours postinfusion. Kruse et al. [32] found that an increase in IL-8 from baseline to 24 hours postinfusion was associated with a favorable depression treatment response in females and an unfavorable treatment response in males (females: *p* = 0.095, effect size (sr^2^) = 0.16; males: *p* = 0.96, effect size (sr^2^) < 0.01).

Park et al. [33] investigated eight cytokine levels and found that levels of IL-6 significantly increased (*p* < 0.001) and sTNFR1 significantly decreased (*p* = 0.006) at 230 minutes postinfusion. Levels changed significantly for IL-5 (*p* = 0.001) and TNF-*α* (*p* = 0.007), but no significant changes were observed from baseline to the later time points. No significant differences were noted for INF-*γ* (*p* = 0.018), IL-10 (*p* = 0.03), IL-2 (*p* = 0.21), or IL-8 (*p* = 0.50). Yang et al. [36] found that serum levels of IL-1*β* showed a significant decrease at 230 minutes and 1 day postinfusion, as did IL-6 at 230 minutes to 3 days postinfusion in the ketamine responder group (*F* = 4.495, *p* = 0.013 for IL-1*β*; *F* = 9.450, *p* < 0.001 for IL-6) but not in the nonresponder (defined as having less than 50% improvement) group (*p* > 0.05). Of the 19 cytokines that were examined in Zhan et al.'s study [34], 14 inflammatory cytokines that differed significantly at 2 week postinfusion (day 26) included GM-CSF, fractalkine, IFN-*γ*, IL-10, IL-12p70, IL-17A, IL-1*β*, IL-2, IL-4, IL-23, IL-5, IL-6, IL-7, and TNF-*α*. These inflammatory cytokines seemed to be downregulated and were significantly lower on day 26 postinfusion than at baseline. Furthermore, the levels of IL-17A and IL-6 were associated with depression symptom improvement on day 13.

## 4. Discussion

Emerging research shows that ketamine efficiently cures both typical and atypical depression symptoms, while the molecular basis underpinning its effectiveness is unknown [20]. Ketamine's likely target in the brain is N-methyl-D-aspartate (NMDA) receptors; by binding to such receptors, it seems to maximize the amount of glutamate in the spaces between neurons [45]. This synaptogenesis influences mood, cognitive habits, and comprehension. Ketamine appears to inhibit glutamate at large dosages, making it an efficient sedative. However, at low levels, glutamate synthesis is increased [46], which could potentially aid in the formation of new interconnections or neurotransmitters between neurons. Whenever individuals are stressed for an extended period, they may lose linkages. Some of these symptoms seem to revert once ketamine is delivered. Therefore, it is proposed to aid in the regeneration of neural synapses [47]. It has been hypothesized that the physiological and pharmacological properties of ketamine may play a therapeutic role in TRD with immediate effects and a decrease in suicidal ideation [48]. Additionally, ketamine treatment could provide a therapeutic option for patients with TRD in managing inflammation-mediated immune cell dysregulation, specifically by suppressing proinflammatory cytokines associated with depression relief [13].

The association between cytokines and their antidepressant effect is multidimensional. Five out of the seven studies examined in this review showed that ketamine may reduce depressive symptoms in patients with TRD with a quick start of effect [30, 32, 34–36]. However, other studies [31, 33] suggest cytokines to be unreliable biomarkers of ketamine's antidepressant effect. These varying findings could be due to differences in the study samples examined: patients with TRD and comorbid pain in Zhou et al.'s study [35] vs. patients with bipolar disorders in Park et al.'s study [33] vs. patients with only TRD in other studies [30–32, 34]. Reports showed that IL-6 levels correlated significantly with clinical response [30, 34, 35]; however, findings of other studies did find an association between the anti-inflammatory and antidepressant effects of ketamine [31–33]. Additionally, Chen et al.'s study [30] showed that patients with TRD in a higher inflammatory state respond to ketamine and other anti-inflammatory agents, while others in a lower inflammatory state did not. Zhou et al.'s study [35] indicated that ketamine had greater effects on modulating inflammation in TRD patients with pain as opposed to patients without pain. Although the anti-inflammatory effects of ketamine seem to modulate proinflammatory cytokines in TRD, its rapid antidepressant effect remains inconsistent. Inflammatory profiles may differ between depressed males and females. Kruse et al. [32] reported that lower baseline IL-8 levels and subsequent IL-8 increase are associated with depression improvement in females, and IL-8 decrease is associated with depression improvement in males. Additionally, Park et al. [33] reported higher IL-8 levels in patients with bipolar disorders (*p* = 0.007) as compared to patients with MDD. However, other studies noted no differences for IL-8 [33–35].

Ketamine seems to aid in the formation of new brain connections, which may aid in the development of resilience and guard against the recurrence of MDD symptoms. Every medicine has adverse effects. Misuse and diversion of these medications are becoming more widespread as their availability increases, and this public health risk undermines their therapeutic benefits. Sanacora et al. [49] indicated that ketamine can quickly relieve depression in people who do not respond well to other treatments. Despite these positive results, we recommend that clinicians consider the risk of ketamine before prescribing it. Zhang et al. [50] reported that the incorrect use of ketamine is a global health problem due to its hallucinogenic effects. The various clinical manifestations and outcomes of inflammation-related depression may be exacerbated in individuals with cytokine dysregulation.

New perspectives have emerged regarding the inflammatory pathways associated with MDD and associated comorbid disorders. Inflammation-related depression caused by cytokine dysregulation may lead to increased pain of varying severity and thus negatively affect depressive symptoms. Comorbid pain disorders are typically linked with depression, and research on ketamine treatment in TRD patients and pain syndromes is scarce. However, the post hoc analysis performed by Zhou et al. [35] suggests that improvements in depressive symptom severity were partly dependent on the improvements in pain symptoms. The authors report the direct (*β* = 2.995, *p* = 0.028) and indirect (*β* = 0.867, *p* = 0.042) effects of the changes of IL-6 levels on reduction in MADRS scores; both were statistically significant. Pain is an independent prognostic factor of depression, and it is therefore suggested that individuals with TRD be screened for inflammatory biomarkers predictive of acute and systemic pain. Because of common neurobiological and molecular pathways, pain and depression are inextricably linked. The pain route uses the same brain structures as the mood control pathway, including the anterior cingulate, thalamus, and prefrontal cortex. Depression and comorbid pain may develop from a shared underlying inflammation mechanism generated by the cytokine storm in individuals with TRD. As a result, early and suitable therapy aimed at proinflammatory cytokines activated by a dysregulated immune system may lower the incidence of inflammation-related pain and depression. Ketamine may aid in treating inflammation-related depression, as well as comorbid pain, which may be exacerbated in the ageing population.

This review identifies the need to examine anti-inflammatory therapeutic strategies targeting various proinflammatory mediators involved in the pathophysiological effect of the ageing process and TRD. While distinct risk variables have yet been identified, it is clear that the ageing population is at a greater risk of immune dysregulation and consequently associated MDD complications. The higher the likelihood of age-related inflammatory disorders, the greater the probability of psychological discomfort and pain. Immunosenescence, a term used to describe the ageing of the immune system, concerns us all. However, depression is not considered a normal part of the ageing process [51]. Age-related changes in immune cells are associated with dysregulation and decline of the immune response. Several dysregulated inflammatory mediators have been observed during immunosenescence and the development of chronic low-grade systemic inflammation, characterized as “inflammaging.” Age-associated immune alterations are characterized as a decrease in the number of peripheral blood naïve cells and increase in the number of memory cells. These alterations, along with inflammaging, are the hallmarks of immunosenescence [52]. There is no cure for immunosenescence and inflammaging; however, they may be managed in many circumstances. Scientific evidence supporting the association between immunological dysregulation and MDD has added to our understanding of inflammaging commonly observed in the ageing population. Aiello et al. [52] proposed that immunosenescence leads to a decline in immune ability causing increased vulnerability to infection and a predisposition to age-related inflammatory diseases. Low-grade inflammatory response, defined as an elevation in serum interleukins and CRP, could have a role in depression etiology and may be an effective indicator of active treatment. For instance, elevated levels of IL-6, TNF-*α*, and their receptors are associated with age-related pathogenesis [53]. According to Singh and Newman [54], older individuals have increased levels of proinflammatory cytokine levels, such as IL-6 and TNF-*α*, compared to younger individuals. Cytokine dysregulation between proinflammatory and anti-inflammatory pathways is a characteristic feature of age-related illnesses and may play an important role in the pathophysiology of inflammation-related MDD.

As indicated in this review, ketamine may have significant antidepressant effects; however, future studies are needed to fully explore its therapeutic benefit. If an individual suffers from TRD, especially with suicidal intent, the potential benefits of ketamine may exceed the potential hazards. Furthermore, ketamine may result in perceptual abnormalities, abdominal discomfort, high blood pressure, and detachment. In clinical practice, any alterations in awareness or detachment are most obvious during the initial ketamine infusion and disappear rapidly afterwards. Ketamine may have additional adverse effects if used regularly or over an extended period of time. Some individuals who have taken a high dosage of ketamine run into the danger of falling into a “K-hole,” which is marked by vivid visual and aural delusions as well as disconnection from reality [55]. Administration of ketamine requires practitioner expertise with treatment-emergent adverse events (TEAEs) and/or related safety concerns. Short-term ketamine treatment in TRD patients may be associated with neuropsychiatric (dissociative, psychotomimetic, psychiatric, and neurologic), cardiovascular, gastrointestinal, genitourinary, and hepatic adverse events [56]. However, the discontinuation rate of ketamine due to TEAEs is moderately low in TRD patients. Ceban et al. [56] suggest that supportive interventions and effective monitoring are sufficient for most TEAEs associated with ketamine treatment.

## 5. Limitations

Systematic reviews provide an objective assessment of the scientific evidence and attempt to minimize bias by utilizing a methodological approach. However, this review is subject to various limitations. Our search strategy was limited to articles written in English, and it is therefore probable that our findings may not represent non-English-speaking settings. However, the studies represented in this review included countries from China, Japan, Taiwan, and USA. To ensure increased reliability in the validity of the methodologies and results, we only included studies that were published in peer-reviewed journals. Thus, unpublished data may be available through other sources that were not included in this review. Also, as in any rapid review, our findings may have been influenced by publication bias. Another methodological weakness of this review is the small sample size which could increase the risk of false-negative results, resulting from studies with conflicting results. Further original studies are needed to adequately advise the clinical practice about ketamine therapy in the management of inflammation-related depression among various age groups—young adults, middle-aged adults, and older adults—with cytokine dysregulation.

## 6. Conclusion

This rapid review identifies the compelling need to investigate several important clinical and public health concerns related to ketamine treatment and inflammatory cytokine markers in TRD patients, preferably by well-designed longitudinal prospective studies and randomized clinical trials. It is well known that depression is a multidimensional disorder, and there is an increasing need to address the biomedical root causes of inflammation-related depression and the immunological response induced by cytokine mediators. These should be looked at as etiological causes of MDD and TRD patients with inflammatory illnesses. Additionally, the pathogenesis of COVID-19 is associated with elevated cytokines such as IL-1*β*, IL-6, and TNF-*α* [57]. With the continuous danger of our ongoing pandemic and adverse mental health effects, clinical comprehension of these biochemical indicators (inflammatory cytokines) and symptomatic results (MADRS/HAM-D) in patients with TRD will aid in the treatment (IV ketamine) approach for this vulnerable cohort (inflammation-related depression). In the meantime, this report contributes to a growing body of information and provides essential guidance to researchers, clinicians, mental health practitioners, and policy-makers.

## Figures and Tables

**Table 1:** Study characteristics for articles reporting on the antidepressant effect of ketamine on inflammatory cytokines in adult patients with treatment-resistant depression.

References (year), country	Study design	Study population and sample size	Age, mean (SD) (y)	Gender differences (female/male)	Dosages, duration, and frequency of IV ketamine	Key findings
Chen MH, et al. [30], Taiwan	Double-blind randomized controlled trial	71 TRD Group 1: 0.5 mg/kg (*n* = 25) Group 2: 0.2 mg/kg (*n* = 23) Group 3: Placebo (*n* = 24)	Group 1: 48.46 ± 11.01 Group 2: 44.96 ± 12.31 Group 3: 48.63 ± 8.12	Group 1: *F* = 21 *M* = 4 Group 2: *F* = 17 *M* = 6 Group 3: *F* = 15 *M* = 9	Dose: 0.5 mg/kg vs. 0.2 mg/kg vs. saline placebo IV duration: 40 min Frequency: Single dose	Biomarkers analyzed: CRP, IL-6, and TNF-*α* Depression scale: (i) A significant dose-dependent decrease in MADRS scores was noted between the three groups across each time point, especially at 40, 80, 120, and 240 min and days 2 and 5 postinfusion (ii) A decrease in TNF-*α* between baseline and 40 min postinfusion was positively associated with changes in MADRS scores on day 4 and day 5 (*p* < 0.05) but not for other days (*p* > 0.05) for the 0.5 mg/kg infusion group Antidepressant: yes, for CRP, IL-6, and TNF-*α* Anti-inflammatory: yes (i) Higher IL-6 (OR: 8.93, 95% CI: 1.16–68.86) level at baseline was significantly associated with a likelihood of treatment response in the 0.5 mg/kg infusion group

Kiraly DD, et al. [31], USA	Open label, repeated measures (ClinicalTrials.gov IDs: NCT01880593, NCT00768430, and NCT00548964)	33 TRD; 26 HC	44.8 ± 13.6 (TRD) 39.0 ± 11.1 (HC)	TRD: *F* = 14 *M* = 21 HC: *F* = 14 *M* = 14	Dose: 0.5 mg/kg IV duration: Not specified Frequency: Single dose	Biomarkers analyzed: broad panel of inflammatory mediators, including IL-1*β*, IL-6, and TNF-*α* Depression scale: MADRS was examined in all patients by treatment response (≥50% reduction in MADRS at 24 h) versus nonresponse Antidepressant: no Anti-inflammatory: yes; however, this modulation is not directly linked to clinical antidepressant effects (i) Levels of all inflammatory cytokines were no longer significantly different from baseline levels (*p* < 0.05) at 24 h postinfusion

Kruse JL, et al. [32], USA	Open-label, clinical trial (ClinicalTrials.gov ID; NCT02165449)	46 TRD	42.3 (11.6)	TRD: *F* = 17 *M* = 29	Dose: 0.5 mg/kg IV duration: 40 min Frequency: Single dose	Biomarkers analyzed: CRP, IL-6, IL-8, IL-10, and TNF-*α* Depression scale: HAM-D scores were collected at baseline and 24 h postinfusion. Response was defined as 50% reduction in HAM-D score from baseline to posttreatment (i) Increasing IL-8 was associated with decreasing HAM-D score in females (*β* = –0.45, *p* = 0.08, effect size (sr^2^) = 0.20), while the inverse was found in males (*β* = 0.43, *p* = 0.02, effect size (sr^2^) = 0.18) Antidepressant: (i) No, for CRP, IL-6, IL-10, and TNF-*α* (ii) Yes, for IL-8 in females only Anti-inflammatory: yes, depending upon gender (i) Lower baseline IL-8 was associated with more favorable antidepressant response according to gender, although the association was not significant (responder status × gender interaction: *β* = –0.36, *p* = 0.096), with evidence of an association in females (*β* = –0.41, *p* = 0.095, effect size (sr^2^) = 0.16), but not males (*β* = –0.01, *p* = 0.96, effect size (sr^2^) < 0.01)

Park M, et al. [33], USA	Double-blind placebo-controlled studies (post hoc analysis) (ClinicalTrials.gov ID: NCT0008699)	49 MDD-TRD; 31 BD-TRD	43.1 ± 12.8 (MDD-TRD) 44.3 ± 12.1 (BD-TRD)	MDD-TRD: *F* = 21 *M* = 28 BD-TRD: *F* = 20 *M* = 11	Dose: 0.5 mg/kg or saline placebo IV duration: 40 min Frequency: Single dose	Biomarkers analyzed: IFN-*γ*, IL-2, IL-5, IL-6, IL-8, IL-10, TNF-*α*, and sTNFR1 Depression scale: MADRS (i) Baseline cytokine levels at 230 min postinfusion were not associated with changes in depression rating scale scores at 230 min (measured as percent change in MADRS score, raw change in MADRS score, or antidepressant response (≥50% MADRS improvement)) Antidepressant: no Anti-inflammatory = yes (i) Levels changed significantly for IL-6 (*F*_3,209_ = 25.51, *p* < 0.001) and TNF-*α* (*F*_3,205_ = 4.18, *p* = 0.007) (ii) BD patients had significantly higher levels of IL-6 and TNF-*α* from baseline through day 3 postinfusion

Yang C, et al. [36], China/Japan	Open label, repeated measures	16 TRD + 24 HV	Not reported	Not reported	Dose: 0.5 mg/kg IV duration: 40 min Frequency: Single dose	Biomarkers analyzed: IL-1*β*, IL-6, KYN, TNF-*α*, and tryptophan Depression scale: HAM-D and MADRS were used to evaluate depressive symptoms at 60 min (baseline) preinfusion and then at 110 and 230 min and days 1, 3, and 7 postinfusion (i) Patients exhibiting ≥50% reduction in MADRS were classified as ketamine responders (*n* = 12), while nonresponders (*n* = 4) were defined as having <50% improvement Antidepressant: yes, for IL-6 Anti-inflammatory: yes (i) IL-1*β* significantly decreased at 230 min and 1 day postinfusion in the responder group (*p* = 0.013) but not in the nonresponder group (*p* > 0.05) (ii) IL-6 significantly decreased at 230 min to 3 days postinfusion in the responder group (*p* < 0.001) but not in the nonresponder group (*p* > 0.05) (iii) TNF-*α* remained the same postinfusion

Zhan Y., et al. [34], China	Open-label, clinical trial (secondary analysis)	60 TRD	34.45 (11.92)	TRD: *F* = 38 *M* = 22	Dose: 0.5 mg/kg IV duration: 40 min Frequency: Six times in a 12-day period (days 1, 3, 5, 8, 10, and 12)	Biomarkers analyzed: GM-CSF, fractalkine, ITAC, IFN-*γ*, IL-1*β*, IL-2, IL-4, IL-5, IL-6, IL-7, IL-8, IL-10, IL-12p70, IL-13, IL-17A, IL-23, MIP-1*β*, MIP-3*α*, and TNF-*α* Depression scale: MADRS was administered at the same time points, and treatment response was defined as ≥50% reduction in MADRS score at 24 h after the final infusion (day 13) compared to baseline MADRS score (i) Remission was indicated by MADRS total score ≤ 10 Antidepressant: yes, for IL-6 and IL-17A Anti-inflammatory: yes (i) 14 inflammatory cytokines, including IL-1*β*, IL-6, and TNF-*α*, differed significantly at 2 weeks postinfusion (day 26) than at baseline

Zhou Y., et al. [35], China	Open-label clinical trial (post hoc analysis)	66 TRD + 60 HC	35.8 ± 11.6	TRD: *F* = 37 *M* = 29	Dose: 0.5 mg/kg IV duration: 40 min Frequency: Three times in a 14-day period	Biomarkers analyzed: GM-CSF, fractalkine, ITAC, IFN-*γ*, IL-1*β*, IL-2, IL-4, IL-5, IL-6, IL-7, IL-8, IL-10, IL-12p70, IL-13, IL-17A, IL-23, MIP-1*β*, MIP-3*α*, and TNF-*α* Depression scale: MADRS was measured at pretreatment baseline, 24 h after each infusion, and again 14 days after the 6th infusion (day 26) (i) Changes in IL-6 levels were associated with reductions in MADRS (*β* = 0.478, *p* = 0.005) scores on day 13 in the pain group Antidepressant: yes, for IL-6 in patients with comorbid pain only Anti-inflammatory: yes (i) 11 inflammatory cytokines, including IL-6, differed significantly among the pain, nonpain, and HC groups (*p* < 0.05)

Abbreviations: BD-TRD: bipolar disorder with treatment-resistant depression; CRP: C-reactive protein; GM-CSF: granulocyte-macrophage colony-stimulating factor; HAM-D: Hamilton Depression Rating Scale; HC: healthy controls; HV: healthy volunteers; IFN-*γ*: interferon-gamma; IL: interleukin; ITAC: interferon-inducible T cell alpha chemoattractant; IV: intravenous; KYN: kynurenine; MADRS: Montgomery-Åsberg Depression Rating Scale; MDD-TRD: major depressive disorder with treatment-resistant depression; MIP: macrophage inflammatory protein; sTNFR1: soluble tumor necrosis factor receptor-1; TNF: tumor necrosis factor; TRD: treatment-resistant depression.

## Data Availability

The raw data supporting the conclusions of this article will be made available by the authors, without undue reservation.
